# The Accessibility of Opioid Agonist Treatment and Its Forced Discontinuation in Swiss Prisons—Attitudes, Perceptions and Experiences of Defense Lawyers in Dealing With Detained Persons Using Opioids

**DOI:** 10.3389/fpsyt.2020.00395

**Published:** 2020-05-14

**Authors:** Anna Buadze, Stephanie Baggio, Roman Schleifer, Eveline Aeberhard, Hans Wolff, Andres Schneeberger, Michael Liebrenz

**Affiliations:** ^1^Department of Psychiatry, Psychotherapy and Psychosomatics, Psychiatric Hospital, University of Zurich, Zurich, Switzerland; ^2^Department of Forensic Psychiatry, Institute of Forensic Medicine, University of Bern, Bern, Switzerland; ^3^Division of Prison Health, Geneva University Hospitals and University of Geneva, Geneva, Switzerland; ^4^Office of Corrections, Canton of Zurich, Zurich, Switzerland; ^5^Psychiatrische Dienste Graubuenden, Chur, Switzerland

**Keywords:** opioid agonist maintenance treatment, prison, qualitative research, defense attorneys, forced withdrawal

## Abstract

**Background:**

Opioid agonist treatment (OAT) is an important pillar in the treatment of individuals using opioids and its continuation during imprisonment is recommended. Despite this knowledge access to and continuation of OAT is still limited in many countries. The forced discontinuation during pre-trial detention can cause severe withdrawal symptoms, which in turn may significantly impair the defendant's ability to exercise granted procedural participation rights. Furthermore, it can be argued that forced discontinuation of a desired treatment represents a form of a compulsory intervention.

**Aims:**

The present study was developed against the backdrop of a recent ruling by the European Court of Human Rights (Wenner vs. Germany). It intended to examine how defense lawyers dealing with detained persons using opioids view and assess the accessibility of OAT in pre-trial detention as well as during imprisonment in different parts of Switzerland.

**Methods:**

Using a qualitative approach, we interviewed 11 defense lawyers from three different cantons of Switzerland with multiple years of experience in providing legal representation to more than 220 defendants using heroin. The interviews were analyzed with QSR NVIVO 11 for Windows. A qualitative content analysis approach was used to evaluate findings.

**Results:**

Defenders who had been exposed to the opioid crisis during the course of their legal career had adopted a positive attitude towards OAT and associated it with a stabilizing influence on their clients, an improvement in criminal prognosis, and a reduction in recidivism. They were generally of the opinion that access to OAT had improved, however identified a considerable variance in different penitentiaries, which were mediated by attitudes of staff and authorities. Based on the assessments of the defense lawyers, it can be estimated that the initiation of OAT especially during pre-trial detention is challenging. The predominant aim of OAT in a variety of Swiss prisons still seems to focus on a discontinuation, mediated by a forced reduction of medication. Some of the interventions reported are not in line with the principle of equivalence and strongly contrast the recommendations of the Council of Europe.

## Introduction

Modern opioid agonist treatment (OAT) with methadone, buprenorphine or other prescribed opioids such as morphine is an important strategy in the treatment of patients with an opioid use disorder (1–4). This form of therapy is associated with an improvement in the individual's health and also leads, among other things, to a reduction in the incidence of HIV and drug-related crime (5–9). Because of these positive effects, methadone and buprenorphine were included in the World Health Organization (WHO) model list of essential medicines almost 15 years ago and have remained on it ever since (10, 11). OAT is also an important pillar in the treatment of opioid addiction in jails and prisons, and various professional medical associations recommend the continuation of therapy during imprisonment (12–14). In this context, it should be emphasized that the overdose-related mortality rate of people with heroin addiction is particularly high after withdrawal under detention conditions and following release without established aftercare (15–17). Despite this knowledge, access to OAT is still limited in many countries, especially for detained persons (18, 19). For example, in September 2016, the European Court of Human Rights (ECtHR) found Germany in breach of Article 3 of the European Convention of Human Rights in denying an inmate access to OAT, even though the applicant had expressed a clear wish to continue with the therapy he had started before he was sentenced to prison. More specifically, the court criticized that authorities had made that decision without having consulted an independent medical expert and without being able to prove the superiority of another form of treatment. This discontinuation of OAT thus amounted to inhuman treatment according to the ruling of the ECtHR (European Court of Human Rights, Wenner v. Germany—62303/13; Judgment of 1 September 2016).

From a medico-legal perspective, the forced discontinuation of OAT during pre-trial detention can cause severe withdrawal symptoms, which in turn may significantly impair the defendant's ability to exercise granted procedural participation rights.

In medico-ethical terms, it can be argued that forced terminations and/or terminations effected against the patient's will of a desired methadone maintenance treatment represent a form of a compulsory intervention (20–22).

Switzerland in particular adopted early on a harm-reduction approach that included low-threshold OAT accessibility, an approach that is currently considered “an ethically legitimate social strategy” (23). Today there are an estimated 30,000 persons or less using heroin in Switzerland, of whom the majority have had OAT on any given day (24). Furthermore, there is even one prison offering not just OAT but also heroin-assisted treatment, which is available to patients who do not respond adequately to methadone and/or buprenorphine and who have been highly dependent for several years (25).

Despite this progress, it is also known, however, that the group of patients with opioid dependence are among those who are particularly stigmatized in a prison setting, and that treatment of substance use is perceived as particularly complicated by prison supervisory staff (26, 27). There is also anecdotal evidence that individual positive or negative attitudes towards the effectiveness of OAT among Swiss prison staff influence its “real life” availability to detained persons, even if access is regulated by high-level prison authorities.

The present study, which was developed in collaboration with the Law Institute of the University of Berne against the backdrop of the ECtHR's ruling, was intended to examine the questions of how defense lawyers representing detained persons using opioids, perceive and experience the work with their clients and how they view and assess the accessibility of OAT in pre-trial detention as well as during imprisonment. This approach was chosen over a written survey of prison authorities in order to reduce the likelihood of socially desirable responses. The aim was therefore not to get a quantitative impression of the quantities and frequencies of a phenomenon such as withholding OAT, or to pillory single detention facilities, but to depict the personal experiences of defense lawyers in dealing with a clientele using opioids. It is precisely the depiction of personal experiences and attitudes that make it easier to understand whether or not there are problems in this context when regarded from a legal perspective, what kind of difficulties these are, and whether they have any wishes for physicians (or any other group of people) (28). The knowledge of the position of the ECtHR and the attitudes of Swiss lawyers can, in the view of the authors, be used for purposes of comparison in other member states of the Council of Europe and thus open up interesting perspectives for an international readership.

## Methods

### Study Design and Reporting

This study was designed with an exploratory qualitative approach and is reported according to the consolidated criteria for reporting qualitative research (COREQ) guidelines (29).

### Sampling Procedure

A mixed purposive and snowball sampling procedure was used for participant selection. We focused specifically on individuals who appeared to be able to provide rich data of the phenomenon of interest, that is, having current personal experience with individuals using substances in criminal justice proceedings. To achieve greater variation of themes and motives, we recruited subjects from three different German-speaking cantons of Switzerland. Furthermore, the sample incorporated diversity with regards to: (a) work experience, (b) legal focus, (c) teaching experience (d), gender and (e) age. The exclusion criterion was unwillingness to give written informed consent.

The research team pursued two strategies to contact potential participants: a.) an opt-in letter (374 words) was sent to certified defense lawyers in the cantons of Berne and Zurich, inviting them to participate. Following a time period of 7–8 days all candidates were then approached by a team member *via* telephone, providing them with more details regarding the research and answering their questions, and b.) individuals who appeared to be especially information-rich were additionally contacted by e-mail or phone and asked to participate; these individuals were previously identified by legal scholars and a high court judge from the cantons of Lucerne and Berne. The latter approach was undertaken to broaden the spectrum and to reach saturation. Saturation is commonly defined as the point when no new themes arise. The subjects provided additional basic biographical data.

### Data Collection and Interview

To ascertain participants' perceptions and experiences in relation to: (1) the legal representation of a clientele using substances, (2) the peculiarities of OAT delivered in pre-trial detention and during the serving of sentences and therapeutic measures, and (3) the further course of their clients' lives after release from prison, we conducted single, semi-structured, in-depth interviews lasting between 60 and 90 min. We used a self-developed and flexible interview guide, which can be found in the appendix. Two female researchers (EA and AB) conducted the interviews. EA was at the time a Master's student at the faculty of law preparing a thesis under the supervision of ML, a forensic psychiatrist and faculty member of the medical school. AB, who was an attending physician at the Psychiatric University Hospital, Zurich with experience in the provision of OAT as well as in conducting qualitative interviews, trained EA and supervised the initial interviews. The research team itself had gathered previous experience employing qualitative research methodology at the intersection of law and medicine. Results have been reported elsewhere (30, 31).

Before the interviews, participants had an understanding that EA had a legal background and that the research represented a collaboration between the Institute for Penal Law and Criminology, the Institute for Forensic Medicine and psychiatric institutions and that the research would address defense lawyers' experiences with clients suffering from opioid dependence.

All interviews were conducted in Swiss or Standard German. Open-ended questions and non-leading probes were used to encourage participants to speak freely and to elaborate on their statements. Paraphrasing and summarizing main points during the interviews helped minimize misunderstandings and clarify ambiguous statements. Interviews were—with the exception of the initial interview—conducted on a one-to-one basis and were digitally recorded. Field notes were taken after the interview

By grounding the questions in participants' practice experiences, and by reformulating the questions, we sought to avoid generalized responses. The location of the interview was chosen by the participants. This was done to create an atmosphere which allowed for eliciting more “private” opinions and experiences. All interviews were carried out at the participants' workplace. There were no repeat interviews.

### Data Analysis

Interviews were digitally recorded using Olympus DS-7000 and then transcribed verbatim into Standard German. Whereas Swiss German is commonly only spoken, Standard German is traditionally used in writing and transcription in Switzerland, which is why all interviews were written down in Standard German using a word processor (Microsoft Word). After removing identifying information, each transcript was assigned a code number. The transcripts were not returned to the participants.

Qualitative analysis of the interview data was done independently, initially by AE, and subsequently for the purpose of this publication by AB and ML. AB and ML analyzed the material blinded as to participant identity, then reviewed the initial categories and themes identified by AE. A comparison thematic approach, identifying common and new themes related to the research aims was used. For this research, the interviews were analyzed with QSR NVIVO 11 for Windows, a qualitative data analysis software (QDAS) (32). This software was used to organize the semi-structured interviews, to set up case nodes, to code emerging themes and to visualize the data. Coding centered on identifying common and unique themes related to the research aims, as well as omissions within the interview transcripts.

**Figure 1:**
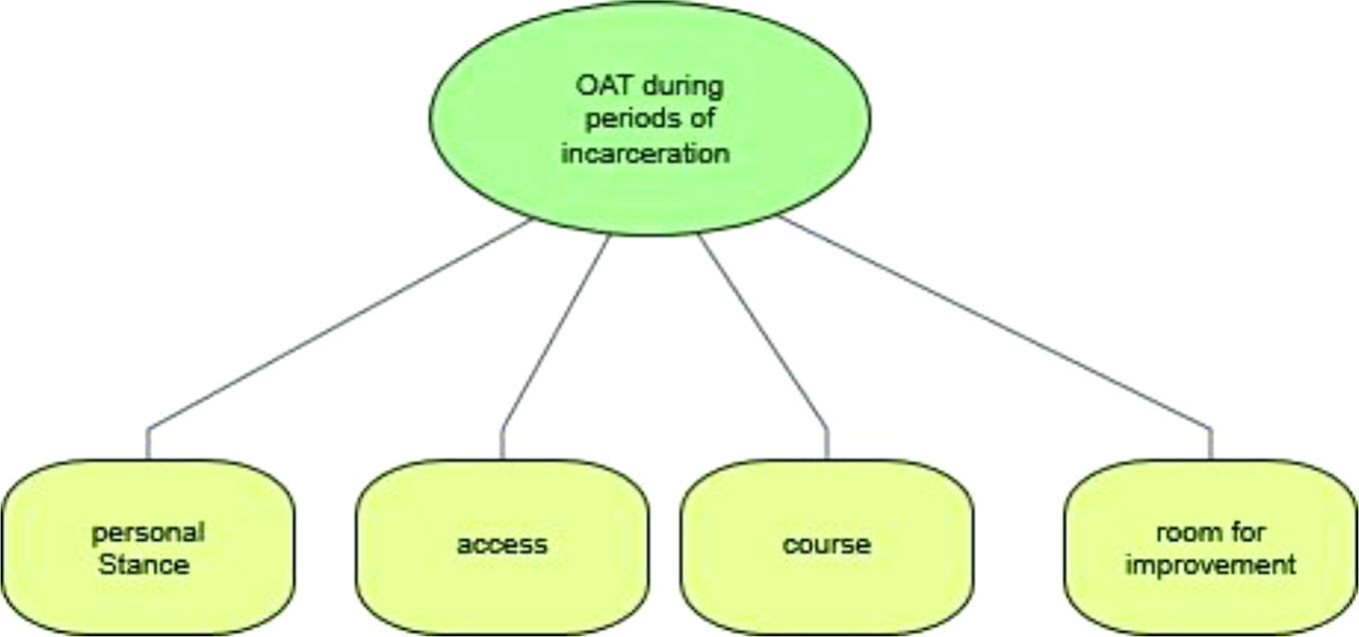
Main categories of lawyers' experience with OAT.

The coding process ensured a systematic, comprehensive and detailed reading of each interview transcript. First, the coders familiarized themselves with the transcripts in order to identify the different subjects of interest. After several interviews had been coded, the categories for the study were redefined, reviewed and revised in a consensual manner at meetings between AB and ML. When there was disagreement regarding the coded material, ML applied the final code. As a result of the coding process and for the purpose of this paper, four main categories were identified and selected: a) personal stance on OAT, b) access to OAT, c) course of OAT, and d) difficulties and room for improvement. An overview of the categories is shown in **Figure 1**.

To illustrate the categories and for reporting purposes, examples of coded quotations were chosen by AE and ML and translated from German into English by ML. Google Translate as well as Deepl were used to support and simplify this translation process. Quotations were then improved by a bilingual German/English speaker (ML) and edited by an English native speaker (Heather Murray) to ensure readability for an international audience.

The quantitative sociodemographic data were evaluated using SPSS version 24.

## Results

### Sample Descriptions

During this study the research team established contact (face to face, telephone or e-mail correspondence) with 58 potential participants. Of those, 47 declined to participate. Barriers to participation included for most lack of experience with defendants using substances, followed by lack of time and lack of interest in the research topic. One of the defense attorneys explained his non-participation in writing (lack of experience), but emphasized the importance of such research projects.

**Figure 2:**
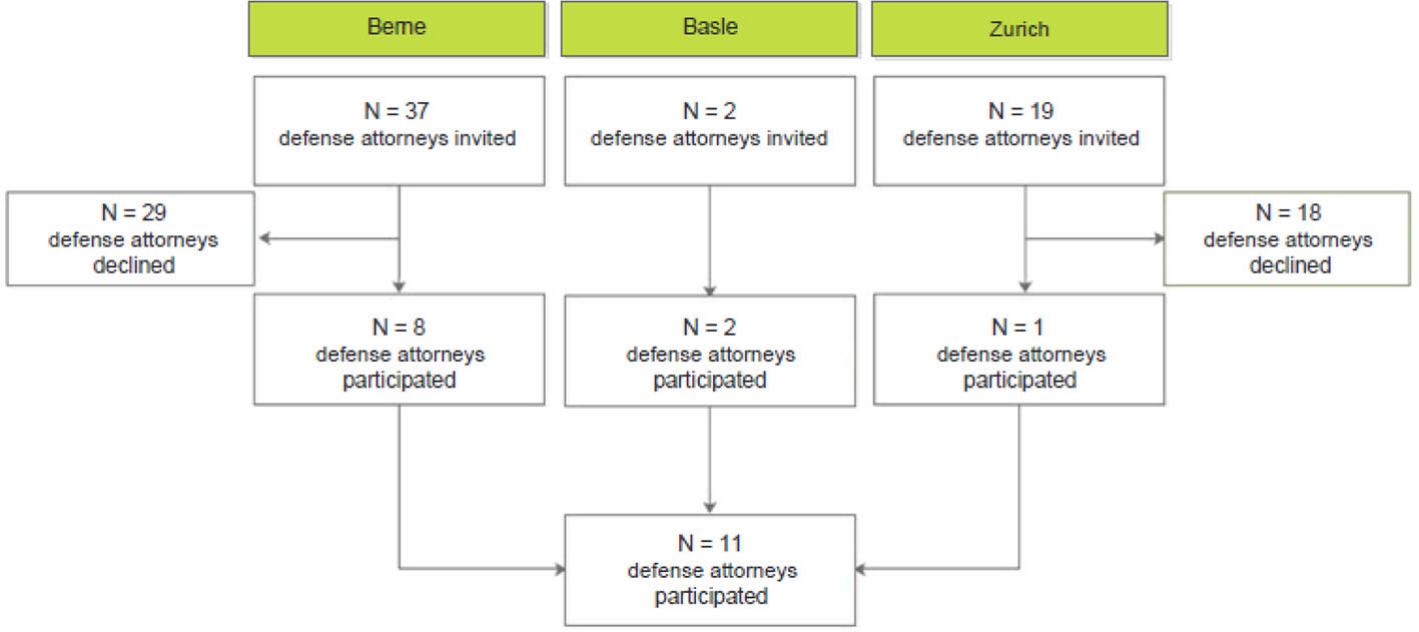
Flow diagram of the study recruitment procedure.

In total, 11 subjects provided their written, informed consent. All completed the interview. None of the participants withdrew their consent at a later time. A detailed flowchart of the recruitment process can be found in **Figure 2**. The sample (n = 11) was composed of a higher percentage of male defense attorneys (81%) than of females (18.2%). The mean age of the participants was 45 years (± 9 years) with an average of 16 years (± 9 years) years passing since taking the bar examination. All had experience in criminal law; additionally, 54.5% had experience in civil law and 9.1% in commercial criminal law (**Table 1**).

**Table 1:** Baseline demographics of participants.

Sociodemographic variables		N (%)	Mean (SD)
Age, Years			45 (9)
Sex			
	Male	9 (81.8)	
	Female	2 (18.2)	
Field of legal expertise			
	Criminal law, yes	11 (100)	
	Civil law, yes	6 (54.5)	
	Commercial criminal law, yes	1 (9.1)	
Certified specialist attorney at criminal law (SAV)			
	Yes	4 (36.4)	
	No	7 (63.6)	
Time since accreditation as certified specialist at criminal law (SAV) year			
	2015	3 (27.3)	
	2016	1 (9.1)	
	2017/2018	1 (9.1)	
	Unknown	6 (54.5)	
Time since bar examination, years			16 (9)
Teaching experience			
	Yes	3 (27.3)	
	No	8 (72.7)	
International work experience			
	Yes	2 (18.2)	
	No	9 (81.8)	
Work experience canton Bern			
	Yes	11 (100.0)	
	No	0 (0.0)	
Work experience in cantons other than Bern			
	0	2 (18.2)	
	1–5	5 (45.5)	
	>5	4 (36.4)	
Current personal experience with individuals using substances			
	Yes	11 (100.0)	
	No	0 (0.0)	

**Figure 3:**
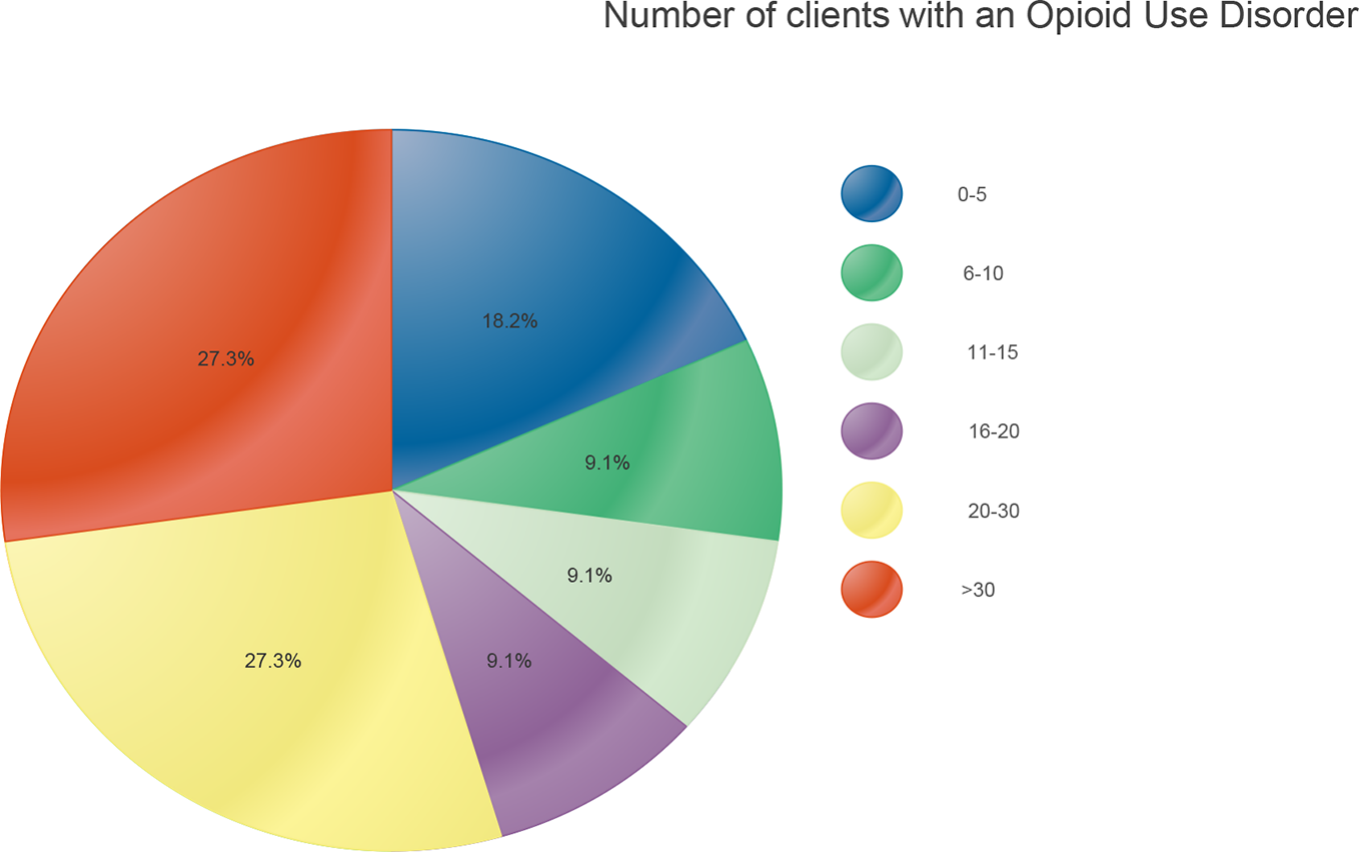
Previous experience with clients using opioids.

Over the course of their career, about half of the defense attorneys interviewed had personally represented more than 20 clients using opioids. Just over a quarter had represented 30 or more such individuals, while a fifth had represented fewer than five clients with such a disorder (**Figure 3**).

### Personal Stance Towards Opioid Agonist Therapy During Times of Detention

**Figure 4:**
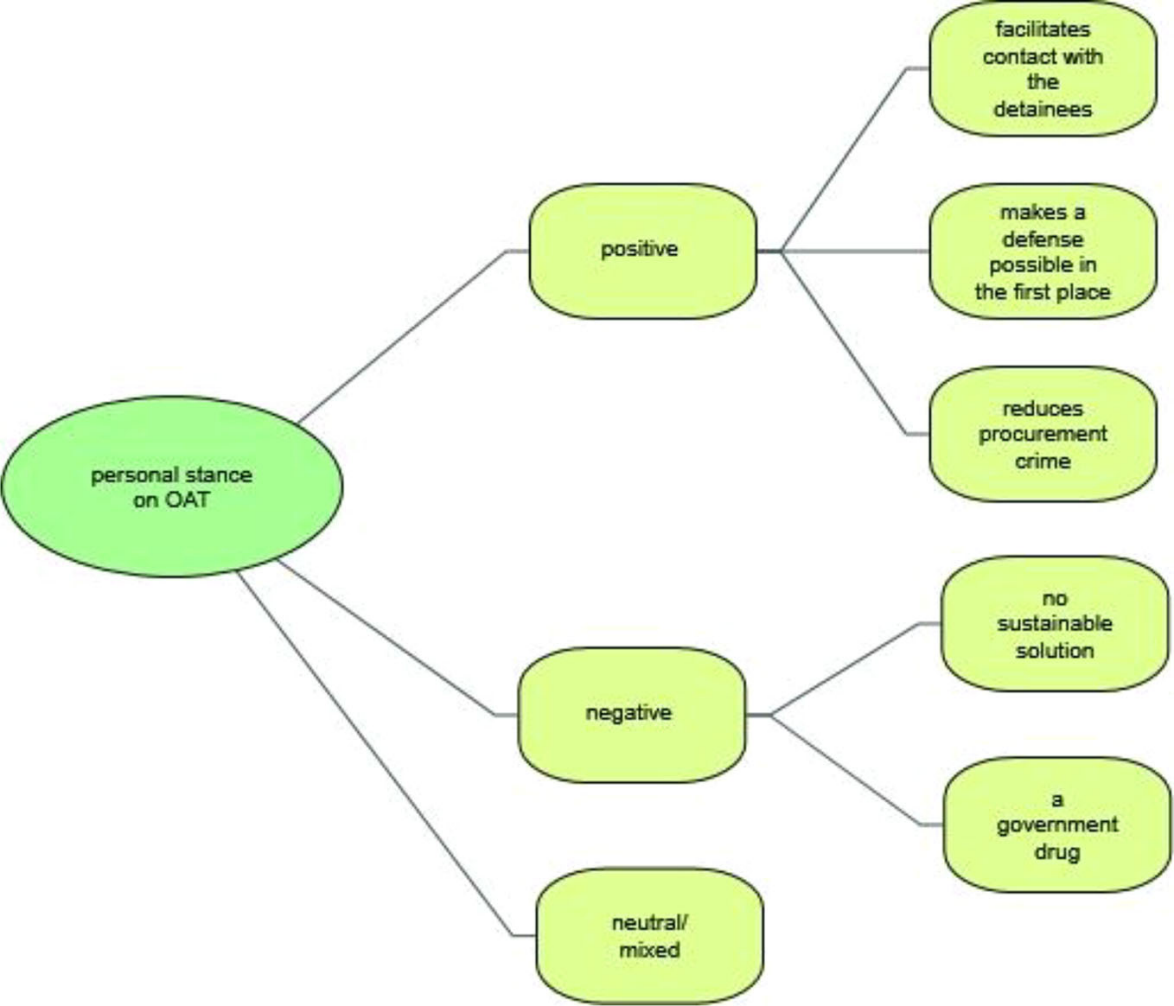
Personal stance towards opioid agonist therapy during times of detention.

One of the first themes to arise was the value that participants accorded to OAT for their legal practice, specifically when dealing with clients using opioids during the early stages of legal proceedings, for example, at times of questioning by the state prosecutor (**Figure 4**).

The defense lawyers interviewed in the context of this study had developed—almost without exception—a positive attitude towards OAT over the course of their legal careers. They perceived it as a “necessary” and “good thing”. Many substantiated this with their own case experience, in which they had come to the conclusion that it was OAT that made defense possible for clients with heroin addiction in the first place.

*“I think it is a very important instrument—a very important instrument especially for the people concerned. But of course also for us as defense lawyers, for us as law enforcement authorities, if you want to include the public prosecutor's office or the court, because, um, you can work with them, let's say in a decent, um, relationship—in a more humane relationship with them. Because in the end you have to talk to them, you have to question them, yes, you have to work together and that of course makes it easier if someone does not—I say it in the vernacular—need to go “cold turkey”*.

And just, yes, somehow the person must be able to live (through that) and must not be oppressed or put under so much stress, because this is bordering on torture, basically, if someone is taken directly in from the streets and then simply put on a cold withdrawal.”

Attorney 10

Multiple times the lawyers also cited a favorable influence on the legal prognosis and recidivism as well as a reduction in procurement crime as reasons for their positive attitudes. A few skeptical comments questioned whether OAT was really a sustainable or long-term solution for those affected.

“It is something very important, very central, um, especially in the area of rehabilitation, improvement of legal prognosis, very important, a very central topic. Almost more important than the penal system itself.”

Attorney 04

There were also highly distinct positions with regard to its classification as a “government drug” in the sense that the state steps in and acts as a “drug-dealer”. Although some expressed the view that this label was “formally” correct, it was the predominant opinion that it is a “superficial contemplation”, a “political statement that has little to do with practice” and “populist nonsense”. Instead, OAT was considered a “pragmatic, economic approach” that helped to steer “addiction in an orderly fashion” and that one had a “social responsibility” towards those affected.

“Yes, that may be true at first sight, but the question is different, I mean what—what costs the state more, if you don't offer substitution afterwards—and the people just fall back into the procurement crime and then you re-examine it…—uh, again conduct criminal proceedings etc. and so on. Then in the end it costs the state a lot more than if it offers substitution and the chance to get these people off the streets, away from drugs. I think the state gets a lot more out of it, so—it may be true, but it is certainly well invested money and the bottom line is that the state certainly saves a lot of money if it offers this possibility.”

Attorney 06

“Substitution—is a controlled release of substance for someone who has an addictive disorder; for me this is nothing other than a treatment (…) analogous to if someone, um, has cancer or, um, I don't know, a migraine—a strong migraine, or–or–or, um, strong pain after surgery, then they also, um, get, um—then they also get strong painkillers, which can also be opiates in some circumstances … and nobody would think that they are on drugs at the expense of the state. So I think that's populist nonsense.”

Attorney 07

### Access to OAT During Times of Detention

**Figure 5:**
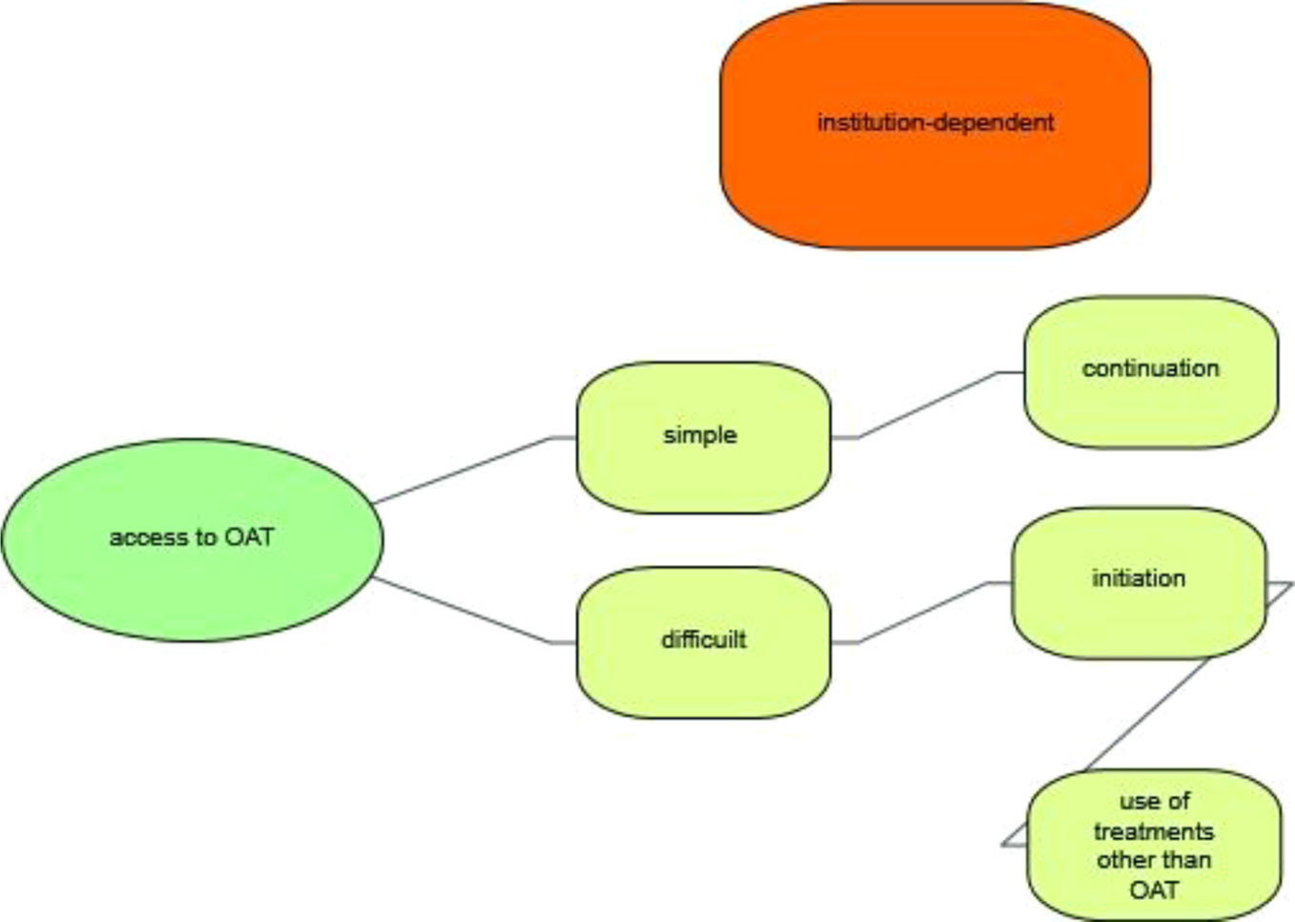
Access to opioid agonist therapy during times of detention.

Participants stressed that there is a considerable variation in the accessibility of OAT in the different penitentiaries (**Figure 5**). The interviewees described institutions in which access to OAT was made either easy or challenging at all stages of criminal procedures, i.e. during police custody or in pre-trial detention, when serving custodial sentences in secure prisons or in the context of therapeutic measures.

Factors that influence accessibility are—in the eyes of the interviewees—the directorate of the detention institution, the health service staff, the medical doctor on call, and the public prosecutor's office.

“Region B. very difficult. So it also depends insanely on how it is run, how the prison director looks at it, how the health service is organized, how easily the prison doctor is reachable, whether he is interested or not, so it also depends on the people.”

Attorney 10

“I've just had a case, maybe half a year ago, there is one, um, a long-time drug addict, who is an addict—he's perhaps fifty today—for 30 years, has also always refused therapy, and he was, um, recently arrested again, and there it was like that, that he demanded the substitute for himself after the arrest, because he did not want a cold withdrawal, and that was denied him in a first phase, however. Um, so the doctors started to give it to him first, and then the public prosecutor intervened—the public prosecutor's office—and then they, um, ver…—didn't give it to him anymore, and then he—he went in—on hunger strike, so he simply didn't accept any more food until they gave it back to him. After three or four days they gave it back to him, maybe faster—I don't know how long it went on, in any case he went on strike and he got the substitute back.”

Attorney 05

Their own influence on prescription practices in individual cases was perceived as extremely low.

“But intervening there as a defender—that is … phew … I mean, in the end they have to—I mean that's, prison is prison, that's the way it is, but they can - they can report their concerns or their wishes or their suggestions to the staff and—and that's it. In the end it's a medical problem and it's not up to the lawyer to say, um, it's still, why are you decreasing the medication? (…) At most, I told clients you have to talk to the doctor, you have to tell him that you need more and that's not possible.”

Attorney 01

Eight out of eleven defense attorneys reported that the OAT offered to detained persons was insufficient for example in respect to dosage, particularly under pre-trial detention conditions - a situation that was experienced as particularly difficult. Some defense attorneys had observed that access to medication was restricted specifically to obtain a confession.

“Especially the police. Just that they delayed [access] to it, that they also say, yes, I know, you are now in “cold turkey”, but now we do this questioning first and then we see if we can organize something, but now we have to talk to you first. And for me this really borders on torture. They don't say: you, if you don't make a statement, then you just don't get anything. They don't say it like that, of course, but they say, look, now we have to do all this first and then we can see if you can get something afterwards, but first we have to make a few phone calls (…) A little in that style, and I have to ask myself what is more urgent now, the health needs of this person, or getting some statements, which will be put in some files and maybe a week later be read by the prosecutor. (…)”

Attorney 10

However, the situation is perceived as being less acute since the opioid crisis has subsided. The defense attorneys distinguished relatively dichotomously between the continuation of an OAT started outside the prison and the initiation of a new treatment. While the former is possible in most prisons, the latter has become almost impossible.

“If they didn't have that before, they had big problems. And uhm, I don't remember when - methadone and heroin maintenance were introduced in prison or in detention. I can't remember when, but since then there were no problems for those who had already been attached to a program before, i.e. outside, they usually had no problems with the continuation of the substitution treatment in detention.”

Attorney 08

In this context, comparisons were also made with prescriptions for other drugs. In particular, the continuation of methylphenidate is perceived as even more difficult as obtaining OAT.

“At least I never got a letter from prison, I'm not getting my methadone or … that was somehow never an issue. Then the public health officers were involved and that was always actually—actually no problem. Quite different with other drugs, right so if someone said I need e.g., um, Ritalin! Oh well, because of ADHD—then it was always a “shit pile”. (The prison doctor said): Ritalin, uh-uh no and all that! No, you don't need that and so on. Do you have a prescription from the doctor who's treating you and so on? And then the client had to call his family and when he finally had it, then the doctor said, no, the last prescription was three years ago. Ritalin is never—it is much more difficult than methadone.”

Attorney 06

In addition, respondents divulged that clients were not treated with OAT after arrest, despite a reported heroin addiction. Instead, withdrawal symptoms were treated with other substances such as benzodiazepines. Clients had complained to their lawyers about this on several occasions.

*“It is not a substitution in itself, but it looks rather as if sedatives, sleeping pills are administered, not necessarily methadone. Now also in a concrete case, I have a client in, um, pre-trial detention. She does not get methadone, but Valium^®^, Stilnox^®^, sleeping pills. She does get drugs, but she does not get a substitution treatment*.

But I know from other clients that I've had before that this is not really a substitution treatment, but more a symptom treatment. Pain—they try to, uh, to suppress pain, and they give sleeping pills, so that they can sleep. Obviously not enough from the client's point of view.”

Attorney 07

### Course of OAT During Times of Detention

**Figure 6:**
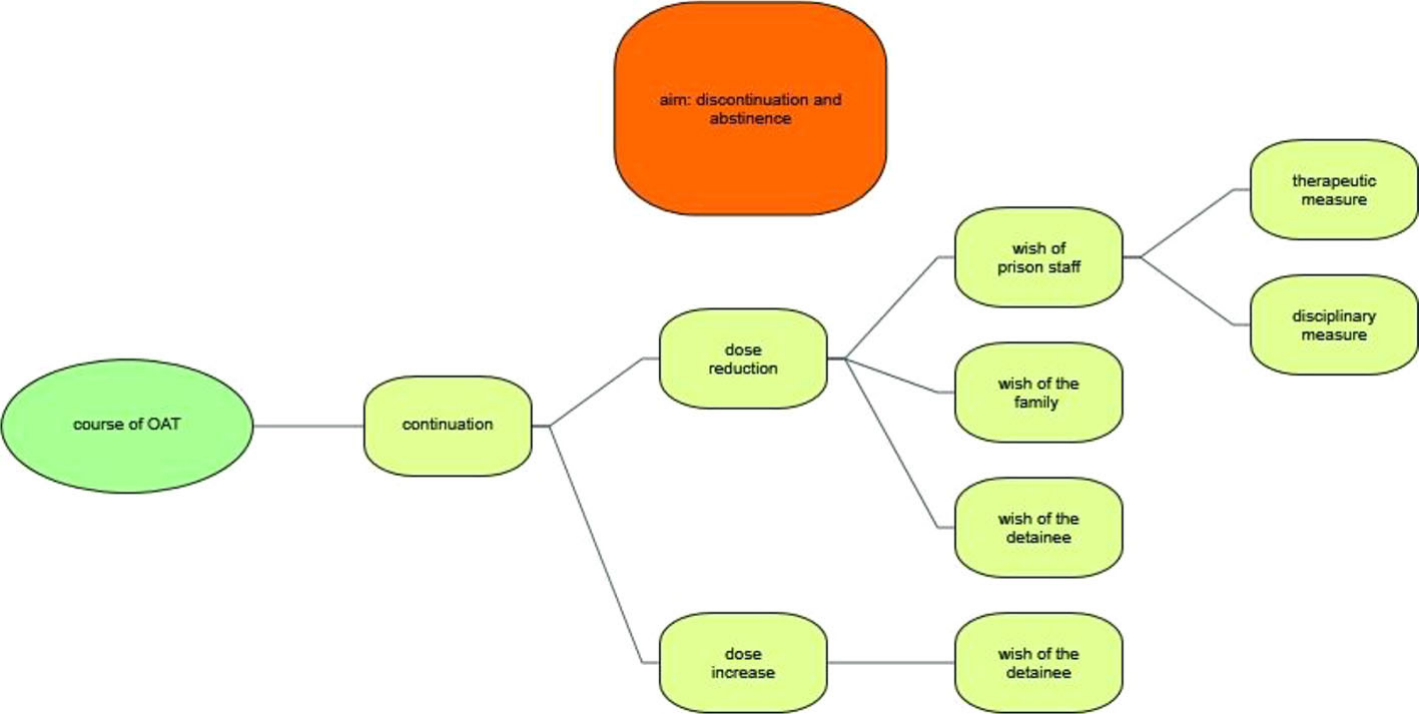
Course of opioid agonist therapy during times of detention.

From the point of view of the lawyers interviewed, most institutions aim to discontinue OAT and to induce long-term abstinence, especially after completion of the main trial (**Figure 6**).

The majority of respondents was in accordance with this therapeutic aim and advised their clients in this direction. This was particularly true when the clients were sentenced to a therapeutic measure according to the Swiss Criminal Code.

“That is actually always the aim, unless it is a very long-lasting substance abuse with a long history and one says, yes, it is rather the aim to substitute. But in most cases it is first and foremost the aim to achieve complete abstinence.”

Attorney 04

“Actually, I think it's right that you don't just provide a substitute for anyone who—anyone who wants to or is involved in a drug-related criminal case as the accused. I think maybe you should try to find your way back to abstinence with those people—if the addictive behavior is not so deeply ground in—to treat and support them.”

Attorney 08

In order to achieve this aim, OAT is usually tapered out during the prison stay. According to the interviewees' experience, reduction steps are initiated and driven by prison staff or legal authorities.

“But of course the authorities or the doctors are pushing a little here and there and they say, so, now let's try again, we'll take a little more away … reduce … I have the feeling that the doctors and authorities are aiming for those dose reductions (…) And I don't think it's a «request show», either.”

Attorney 01

In addition to a dose reduction driven for therapeutic motives, the interviews also showed that dose reduction is used as a disciplinary measure.

“For example, as punishment and that's of course a huge problem for these people, um, if they get that as punishment, because then—then they riot even more, they can't understand it at all, they're not rational, or, they don't see a causal relationship.”

Attorney 03

Another major factor in dose reduction is the family's desire to encourage detained family members to discontinue opioid maintenance treatment.

“And, so then there are often the parents behind it and they say: Now look, now you are in prison, now you can get away from this [methadone]stuff, so that you're done with it once you get released.”

Attorney 01

Ultimately, the detained persons themselves push ahead with the discontinuation of OAT, when they perceive this as an opportunity to live a life free of any substances.

“And the clients say: No, this is the moment now. I have been taken in by the police. This is my chance to stop using and I want to do it cold turkey. Bam. And then I go in and I talk to them and ask what they need and then there's usually a little bit of back and forth. And they ask me for as many cigarette packets as I can bring, or for a couple of kilos of chocolate and then they basically self-medicate, so to speak.”

Attorney 10

On the other hand, an increase in the dosage is always driven by the detained persons. However, considerable resistance by staff is described in this context. In no case did the defenders report that the dose had been increased because, for example, there had been co-consumption.

*Well, in one case I can remember how they had to go back up after a reduction of methadone, because somehow it just didn't work out at all for the client. But as a rule of thumb, I think that they want to come down with these substances*.

Attorney 01

### Room for Improvement

**Figure 7:**
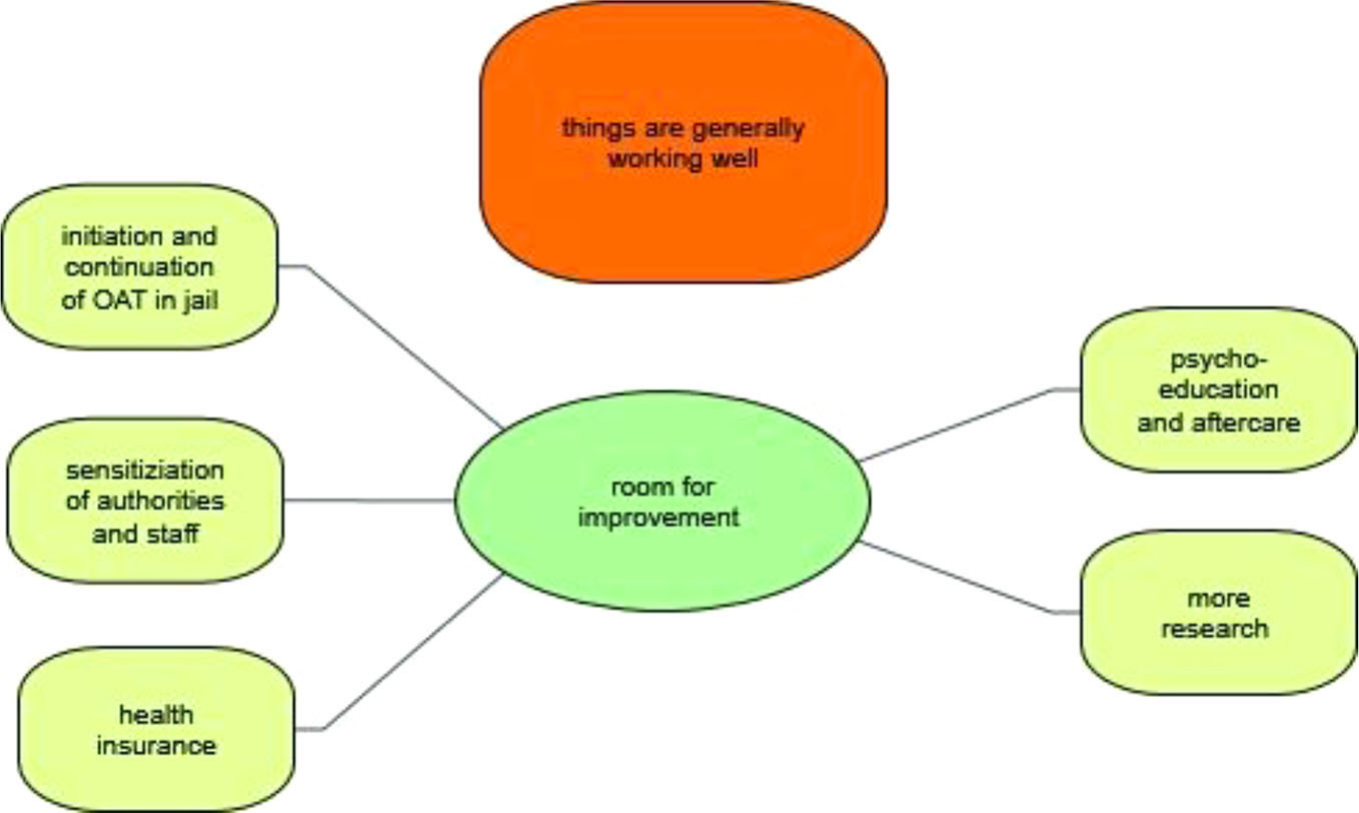
Room for improvement.

Despite shortcomings, the defense lawyers interviewed were generally of the opinion that there was relatively little need for action with regard to OAT in Switzerland (**Figure 7**). The majority of those interviewed had come to this conclusion because they had received few(er) complaints from their clients and had generally noticed a decline in clients with opioid misuse and/or drug-related crime. In this context, a comparison was also made with neighboring countries and the USA. Some of the Swiss defense lawyers were well informed about the course of the opioid crisis there.

“But otherwise I actually have the impression that the level, as it is now in Switzerland, is quite exemplary in comparison with other countries, yes.”

Attorney 09

Against the background of the problems reported with access to OAT under pre-trial conditions, it was not surprising that most defense lawyers mentioned the need for improvement in this area. In this context, it was emphasized that an OAT should be started as quickly and with as low a threshold as possible. Occasionally, the discontinuation and tapering out of OAT during transition from pre-trial to prison was criticized, but this was then relativized again by the stricter regime prevailing in a closed prison setting.

“Something that could be improved is that if clients are arrested who are acutely addicted to drugs and want methadone substitution, that this could be introduced more quickly.”

Attorney 11

Another aspect that was highlighted was the need to raise awareness among law enforcement officers for the needs of individuals using heroin. According to the respondents, a strong focus should be placed on the younger generation of police officers who did not experience the opioid crisis in Switzerland in the early 1990s. Here a clear determination of the criminal defense lawyers became noticeable to preserve what had been achieved since then.

“But I believe that this is an issue on which we must continue to be sensitive. We also need to sensitize new police officers, people in law enforcement, in particular, and say, be careful, you have to deal with [opioid-using individuals] differently. You have to be careful at the very beginning. There must be procedures in place that run virtually automatically. Even in the first few hours you need someone to ask about people's needs. And this person needs to find out whether the defendant is enrolled in an existing program or whether a new one has to be started.”

Attorney 10

A need for improvement was also advocated in the area of psycho-education and aftercare after release from prison.

“If you can arrange for that during the time served, so that they can enter a program immediately after release, then that would be quite meaningful.”

Attorney 01

Those defense lawyers who had clients who had died of an overdose following prison release had begun informing their clients themselves about the loss of tolerance after withdrawal.

*And that's something I always tell clients when they were abruptly withdrawn in pre-trial detention. You really try to sensitize them for the fact that this is really dangerous when they get out and—and just start again*.

Attorney 05

At the level of society as a whole, it was also desirable that information about the risks of drugs should be provided earlier and more intensively during adolescence. There were also isolated calls for research into better substitution agents, the side effect profile of which should be more favorable. The expectations and wishes towards the development of new drugs for the pharmaceutical treatment of opioid using individuals went beyond what is understood as opioid agonist treatment from a medical perspective.

“Yes, there is a need for action in the sense of - so what I would like to see is, to push research harder, to develop pharmaceuticals that have fewer side effects, that do not result in another dependence.”

Attorney 09

In addition, the need to secure access to health insurance services for prisoners was emphasized, as defense lawyers felt this provision was under increasing political scrutiny.

## Discussion

In the present qualitative study, we investigated how Swiss defense attorneys view OAT, its accessibility and course in pre-trial detention as well as during imprisonment in different parts of Switzerland.

Our results indicate that defense attorneys working closely with a clientele using opioids view OAT as a “valuable, pragmatic and economic” medical intervention. In their understanding, the treatment allows easier contact with their clients, as well as a more effective defense and goes hand in hand with an improved legal prognosis. This finding is in line with the research on OAT's effectiveness, but contrasts with reports from the United States claiming that “few defense lawyers understand the literature, science and research that supports their arguments” for OAT in the proceedings before drug-courts (33). From their statements in the present study it could be inferred that Swiss defense lawyers had experienced the difficulties of unaccompanied detoxification in the sense of “cold-turkey” first-hand. Negative effects of detoxification are “often associated with a variety of unhealthy behaviors” on re-entering OAT after prison release and have been documented elsewhere (34, 35). Jonsen and Stryker warned that detained persons who ease their withdrawal symptoms without formal support also put themselves at increased risk by using drugs available in prison or buying medications from other inmates (36).

Defense attorneys were generally of the opinion that access to OAT either with methadone or buprenorphine, and in some cases diacetylmorphine, in the Swiss penal system had improved over the years. However, they identified a considerable variance in the accessibility of OAT in different penitentiaries. Unexpectedly this heterogeneity was not limited to police-custody or pre-trial detention, but was also described for institutions carrying out therapeutic measures according to Art. 60 of the Swiss Criminal Code. The aim of this therapeutic measure is to reduce the risk of reoffending by an delinquent dependent on psychotropic substances, whose offence was linked to this dependency, and of whom it can be said that, with treatment, the risk of further such acts in connection with this disorder can be reduced. The treatment is provided in specialized inpatient institutions, or, if necessary, in a psychiatric clinic. The unavailability and/or forced discontinuation of OAT in institutions carrying out Art. 60 measures strikes one as especially problematic, considering that these institutions involve medical professionals in the treatment process, who should know about the efficacy of this form of therapy while the unavailability cannot be proven by our methodical approach, the interviewed individuals perceived the availability of OAT as not sufficient. This should be subject to further research.

This significant heterogeneity in access to OAT has also been reported from 18 other European countries, including Germany (37). In these countries, too, it can be seen that although OAT is nominally accessible, its actual implementation varies between prisons even within the same jurisdiction (38).

Apart from the variance in accessibility, it became apparent that the continuation of an OAT started outside the prison was considered to be significantly easier than the initiation of OAT in pretrial detention or during a prison term. This phenomenon has previously not been reported for Switzerland and, given the highly successful four pillar drug policy implementation (39, 40), was rather surprising. This restriction concerns in particular foreign nationals who are not permanent residents of Switzerland, who are in detention and thus cannot prove that they have taken part in an OAT, for example in their home country. The discussion of this problem must remain open and was not within the scope of the present study. From a scientific point of view, there is strong evidence that prison-initiated methadone maintenance leads to an increased likelihood of entering treatment post-release and is associated with less use of heroin after release, other opiates and injection drugs (41).

A recurring motive in this context was that, instead of opioid-agonist medicine, other drugs were prescribed to cope with symptoms of opioid withdrawal. Benzodiazepines were mentioned here in particular, but also non-benzodiazepine sedatives and hypnotics. Although not in line with current international recommendations for the management of opioid withdrawal symptoms, which suggest tapered doses of opioid agonists (42), this course of action has been reported from correctional institutions elsewhere (34). A major concern regarding benzodiazepine use in individuals using opioids is their potential contribution to an increased opioid‐related mortality as well as the development of a subsequent long term misuse of this substance (43). Our study revealed that perceived difficulties with non-opioid agonist medication like benzodiazepines used to mitigate withdrawal symptoms are discussed and debated among defense lawyers and clients, but that these discussions are constrained by the fact that defenders assess their influence on prison staff as being extremely low.

From the point of view of medical ethics and the rule of law, the perception of some defense lawyers that access to opioid-agonist medicine is restricted in order to increase the defendant's willingness to testify is worrying. Specific examples were given in particular in connection with the placement of individuals using opioids in police custody, i.e., in the early stages of criminal proceedings, since police custody is (according to the Swiss code of criminal procedure) limited to 24 h. From a medical perspective, it should not go unmentioned that the discontinuation of methadone represents a considerable psychological burden and has been considered a trigger for suicidal behavior in pre-trial detention (44, 45). Naturally, a qualitative study cannot give an assessment of the frequency of a phenomenon. However, future studies, using a different methodological approach, should review this aspect.

Surprisingly, the defense attorneys were under the impression that authorities in most correctional institutions still aimed in the long run at a discontinuation of opioid-agonist medicine and recommended this over a maintenance approach to the inmates. Interviewees shared this point of view, despite their overall positive stance on OAT—a finding that underlines that the stigma associated with this treatment approach is particularly strong (46, 47) and further education and training of legal actors may be necessary

In view of the positive results that have been published with regard to the introduction of OAT from selected Swiss penal institutions (48, 49), this finding underlines considerable heterogeneity, not just in terms of access, but also in the course of OAT and implies that “access” is not synonymous with “continuation of treatment”. The present study cannot answer the question of why some Swiss institutions are apparently enforcing or at least promoting the discontinuation of opioid-agonist medicine. Various studies have shown that the mortality rate of detained persons using opioids immediately after release from closed prison without OAT is increased and remains at an elevated level (17, 50, 51). The elevated risk of fatal overdoses in this population has been linked to the loss of tolerance and is estimated to be three to eight times greater than that during other periods at liberty (52). Similar findings were recognized early on in Switzerland (53) and influenced the development of recommendations by the Swiss Society of Addiction Medicine (SSAM) and the Federal Office of Public Health (FOPH) that list the discontinuation of OAT as only one of four treatment options, explicitly stating: “As OAT is also one of the preventive measures against overdose deaths, it is preferable that the withdrawal of the opioid-agonist medicine should take place *after* discharge” (54, 55). Stöver et al. have commented on barriers to implementation of OAT in prison and identified for example a “poor understanding of opioid dependence as a chronic and recurring disease”, a “mistaken belief in the benefits of abstinence for drug users”, “socioeconomic reasons”, a belief that OAT is not compatible with the concept of prison as a “drug-free zone”, a belief that this form of treatment undermines efforts to reduce drug supply and amplifies “diversion” as such (56). It is conceivable that a number of these factors still play a role in the Swiss prison system, although Switzerland played a pioneering role in the implementation of OAT.

The majority of participants described a forced tapered withdrawal from methadone and/or other agonist medicine that was initiated and driven forward by prison staff or legal authorities and identified accelerated reduction leading to suboptimal dosage levels as a major concern for their clients. Some statements suggest that selected penal institutions show very little flexibility in dose adjustments, that clients are not always involved in the decision-making process and that dose reductions are carried out explicitly against the wish of the detained person. This indicates that in some Swiss prisons federal regulations might not be fully implemented, since, for example, the above-mentioned FOPH recommends that: “Every person using opioids should have an individualized treatment that is tailored to their needs and is adapted to the clinical course, personal motivation and legal circumstances.” It further advises those involved to “Consider the possibility to revise the decision as to dose change, within the framework of the requirements of the respective institution regarding prescription or clinical evaluation (weekly doctor's visit).”(54).

The importance of OAT for rehabilitation and relapse prevention was emphasized several times, thus becoming a recurrent motive amongst defense lawyers. One of the respondents remarked that OAT is not easily granted in one of the secure prisons carrying out sentences of indefinite incarceration because “the idea of rehabilitation does not play a role there”. This illustrates the extent to which staff attitudes also influence access to medically indicated treatment—a result that is consistent with international reports on the accessibility of health services to inmates (57–59).

It should not go unmentioned that, in the opinion of the defense lawyers, some detained persons are using their stay in prisons to end OAT on their own initiative. The detained persons are supported in this by family members. Both aspects were reported in a similar form from other countries (34).

For some of the interventions reported, it must be noted that these are not in line with the principle of equivalence, that is, prisoners shall have access to the health services available in the country without discrimination on the grounds of their legal situation and conflict with legal requirements on a federal as well as, in some cases, on a cantonal level (60). In particular, the above-mentioned forced discontinuation of OAT against the will of the detained persons, but more so the termination of OAT for disciplinary reasons—as described by some defense attorneys—would be unethical from a medical point of view and contrasts starkly with recommendations laid out by the Council of Europe and the perspective of the European Court of Human Rights as reflected for example in its ruling Wenner vs. Germany.

Surprisingly, the defense lawyers saw little need for action with regard to OAT. Comparisons were repeatedly made with the situation in past decades, when accessibility was even more difficult. The defense lawyers had also subjectively noticed a decline in the number of clients suffering from heroin addiction, which is why the strategies applied were assessed positively. In this context it can be pointed out, that current data shows a stable prevalence of problematic heroin use with a sharply decreasing incidence (61, 62). The (subjective) experiences of the attorneys also coincide with reported declines in drug-related crime and suggest that the success of Swiss drug policy is palpable on the practical everyday level of criminal proceedings (63–65). However a decrease in incidence of problematic heroin use can and should not justify poor OAT practices.

Comparisons were also made between the accessibility of opioid-agonist medicine and stimulant medication such as methylphenidate, which was considered even more difficult to obtain. Data on the accessibility of methylphenidate in Swiss prisons does not exist, but a recent study suggests that only a third of inmates who are diagnosed with ADHD receive stimulant treatment (Baggio et al., under review).

The aspects that needed—in the eyes of the interviewees—improvement were not surprising and were largely complementary in nature. Defense lawyers emphasized the necessity for a more low-threshold approach to initiation of OAT during pre-trial detention, were in favor of a smoother transition from pre-trial to prison, demanded a higher degree of sensitization of authorities and staff to the needs of detained persons using opioids and advocated for improvements in their psycho-education and aftercare after release from prison. Regarding the lobbying for improved psycho-education, it may be noted that defense lawyers themselves had begun to advise their clients on drug related health-issues, e.g. by pointing out the dangers of overdoses after release from prison, and were thus, without classifying their strategies as such, educationally active and applying, unbeknownst to themselves, a harm reduction strategy (66). This observation underlines the importance of imparting knowledge to legal professionals (31). Defense attorneys' concerns about detainees' access to medical care are not unfounded. Although the directives on the provision of medical heath care for persons in detention stipulate clearly that medical care has to be accessible at a low-threshold level and should in principle be free of charge, except for some minor copayments, reality deviates from this rule. Access to health services for detainees seems to have become more complicated in recent years and physicians have to deal with additional work, such as applying for reimbursement of costs and negotiating with administrative bodies in prison or social services to provide their patients with adequate health care (67).

### Limitations

These results need to be considered within the limitations of the investigation. First, because this is an exploratory qualitative study based on a purpose sampling method, the findings on the personal stance towards OAT cannot be generalized beyond this study sample. However, with regard to the other themes identified, the sample represents a group of defense lawyers with multiple years of experience in providing legal representation to at least 220 defendants using heroin. Second, there are limitations associated with volunteer bias, to which most studies are also susceptible. The main reason for non-participation stated was lack of experience with substance-abusing defendants, followed by lack of time and lack of interest in the research topic. However, other possible reasons could include sensitivity regarding the topic.

Since we only interviewed defense lawyers who had experience with clients using heroin, our findings may not reflect the attitudes of recently graduated lawyers. As exploratory research, this study was not driven by a theoretical framework. Future studies on this subject could, however, use the insights gained to pursue more focused research.

We also recognize that the results may in part be specific to the Swiss legal and penal system. Nevertheless, the literature indicates that similar problems, such as significant heterogeneity in access to OAT, have also been reported from other European countries.

Our findings provide several relevant insights into views held by defense lawyers who gathered vast experience during the height of an opioid crisis and in the following years until today. Most importantly, our findings are based on defense lawyers' own reports identifying a range of experiences. These findings were not limited to predefined experiences, as might occur in a survey-based research. Furthermore, a written survey of prison authorities might have increased the likelihood of socially desirable responses. Such criticism could also have been voiced if the study had focused on the experiences of current or past detained persons using opioids themselves.

### Conclusions

This research gives additional insight into the accessibility of OAT and its forced discontinuation in Swiss prisons as experienced by defense lawyers. Defenders who had been exposed to the opioid crisis during the course of their legal career had adopted a positive attitude towards OAT, and associated it with a stabilizing influence on their clients, an improvement in criminal prognosis and a reduction in recidivism. They were generally of the opinion that access to opioid maintenance treatment in the Swiss penal system had improved over the years, however identified a considerable variance in the accessibility and the course of OAT in different penitentiaries, which were mediated by attitudes of staff and authorities. Based on the assessments of the defense lawyers, it can be estimated that the initiation of OAT, especially during times of police-custody and during pre-trial detention is challenging, especially for clients who have not been enrolled in OAT prior to being arrested. Furthermore, the predominant aim of OAT in a variety of Swiss prisons seems, in contrast to the available medical evidence and the harm-reduction drug policy implemented, still to focus on a discontinuation of OAT, mediated by a forced reduction of medication. For some of the interventions reported, it must be noted that these are not in line with the principle of equivalence and conflict on a federal as well as, in some cases, on a cantonal level with legal requirements, while at the same time starkly contrasting with recommendations laid out by professional societies, the Council of Europe and the WHO. The defense lawyers advocated for improvements in the areas of psycho-education and aftercare after release from prison of detained persons using opioids and, in the perceived absence of these, regularly advised their clients on drug related health-issues, e.g. by pointing out the dangers of an overdose after release from prison. It may therefore make sense, alongside other legal professionals such as prosecutors and judges, to specifically train defenders of a clientele with substance use dependence on harm reduction measures and make relevant knowledge easily available to them.

## Data Availability Statement

Excerpts of the transcripts relevant to the study are available on substantiated request by the corresponding author.

## Ethics Statement

The research was conducted in accordance with the 1964 Helsinki Declaration. Zurich's cantonal ethics committee filed a letter of non-competence, stating no objection and exempting in from in depth-review according to the framework of the Swiss Federal Act on Research involving Human Beings. All participants were assured confidentiality, and gave their written informed consent to the study and, specifically, to the digital recordings of the interviews.

## Author Contributions

AB/EA: Data gathering, evaluation of data, writing. RS: Conception, development of the topic guide, revisions. SB/HW/AS: Writing, revisions. AB/ML: Conception, development of topic guide, obtaining ethical approval, evaluation of data, writing manuscript, revisions.

## Conflict of Interest

The authors declare that the research was conducted in the absence of any commercial or financial relationships that could be construed as a potential conflict of interest.

The reviewer IP declared a shared affiliation, with no collaboration, with several of the authors, SB, RS, EA, AS and ML, to the handling editor at the time of review.
